# miR-758-3p: a blood-based biomarker that’s influence on the expression of CERP/ABCA1 may contribute to the progression of obesity to metabolic syndrome

**DOI:** 10.18632/oncotarget.24314

**Published:** 2018-01-24

**Authors:** Sadhbh O’Neill, Mette Bohl Larsen, Søren Gregersen, Kjeld Hermansen, Lorraine O’Driscoll

**Affiliations:** ^1^ School of Pharmacy & Pharmaceutical Sciences and Trinity Biomedical Sciences Institute, Trinity College Dublin, Dublin 2, Ireland; ^2^ Department of Clinical Medicine, Aarhus University, 8000 Aarhus C, Denmark

**Keywords:** metabolic syndrome, obesity, miR-758-3p, CERP/ABCA1, biomarker

## Abstract

Due to increasing prevalence of obesity, a simple method or methods for the diagnosis of metabolic syndrome are urgently required to reduce the risk of associated cardiovascular disease, diabetes and cancer. This study aimed to identify a miRNA biomarker that may distinguish metabolic syndrome from obesity and to investigate if such a miRNA may have functional relevance for metabolic syndrome.

52 adults with clinical obesity (n=26) or metabolic syndrome (n=26) were recruited. Plasma specimens were procured from all and were randomly designated to discovery and validation cohorts. miRNA discovery profiling was performed, using array technology, on plasma RNA. Validation was performed by quantitative polymerase chain reaction. The functional effect of miR-758-3p on its predicted target, cholesterol efflux regulatory protein/ATP-binding cassette transporter, was investigated using HepG2 liver cells.

Custom miRNA profiling of 25 miRNAs in the discovery cohort found miR-758-3p to be detected in the obese cohort but undetected in the metabolic syndrome cohort. miR-758-3p was subsequently validated as a potential biomarker for metabolic syndrome by quantitative polymerase chain reaction. Bioinformatics analysis identified cholesterol efflux regulatory protein/ATP-binding cassette transporter as miR-758-3p’s predicted target. Specifically, mimicking miR-758-3p in HepG2 cells suppressed cholesterol efflux regulatory protein/ATP-binding cassette transporter protein expression; conversely, inhibiting miR-758-3p increased cholesterol efflux regulatory protein/ATP-binding cassette transporter protein expression.

miR-758-3p holds potential as a blood-based biomarker for distinguishing progression from obesity to metabolic syndrome and as a driver in controlling cholesterol efflux regulatory protein/ATP-binding cassette transporter expression, indicating it potential role in cholesterol control in metabolic syndrome.

## INTRODUCTION

Metabolic Syndrome (MetS) is an ever-increasing concern worldwide due to the increase in obesity and sedentary lifestyles. It is estimated that one quarter of the adult population has MetS [1]. To date there are three main definitions of MetS, set out by the International Diabetes Federation (IDF), the World Health Organisation (WHO) and the National Cholesterol Education Program: Third Adult Treatment Panel (NCEP:ATPIII) [2], that differ in minor details [3, 4].

Obesity is fundamental to MetS as it appears to precede the emergence of the other MetS risk factors [5]. MetS culminates from the clustering of component risk factors including obesity [6]. Individuals with MetS are five times more likely to develop Type 2 diabetes (T2DM) and three times more likely to suffer from cardiovascular disease (CVD) [7]. Furthermore, the development of many cancer types is associated with obesity and MetS. A meta-analysis of 38,940 cases of cancer found the presence of MetS to be significantly associated with liver, colorectal, bladder, endometrial, pancreatic, and postmenopausal breast cancers [8]. As the prevalence of MetS is increasing, the prevalence of these conditions is increasing in parallel. Accurate and timely diagnosis of MetS is, therefore, of critical importance to avoid this increased risk.

Despite the increasing prevalence and thus the socio-economic stresses associated with MetS and its subsequent pathologies, a panel of blood-based miRNA biomarkers for MetS has not yet been established. Mature miRNAs are small ~21 nucleotide long noncoding single-stranded RNAs that are widely distributed throughout all eukaryotic organisms [9]. miRNAs are encoded in the genome [10] and they bind in an anti-sense manner to complementary mRNA. miRNAs function to increase or decrease gene expression post-transcriptionally [11]. A number of miRNAs have been identified that increase or decrease components of MetS, cholesterol and fatty acid synthesis (*e.g.* miR-33a/33b, miR-758, miR-204, miR-106b and miR-200). Some proteins such as adiponectin [12], leptin [13] and human c-reactive protein [14, 15] have been proposed as new biomarkers. However, in reality, in the clinic MetS is diagnosed by assessing insulin resistance, triglyceride levels, high density lipoprotein cholesterol, blood pressure and body-mass index or waist circumference; the latter which some individuals find embarrassing and so avoid being tested. Profiling circulating miRNAs could realistically contribute to a more comprehensive panel of minimally-invasive, blood-based biomarkers for diagnosing MetS. Thus, here we profiled circulating plasma miRNAs from MetS and obese individuals to identify if any miRNAs exist that might distinguish MetS from obesity and that may have diagnostic and functional relevance in the pathophysiology of this condition.

## RESULTS

### Participant plasma specimens

Ninty-nine individuals initially gave informed consent, however 25 withdrew consent prior to screening. A total of 74 were screened, from these 74 there were 11 dropouts (screen failures, 4 problems with IV access, 2 personal reasons, 1 due to disease exacerbation). A total of 63 began the study, however due to technical difficulties no intravenous access was obtained for one of the participants. Fat biopsy specimens were not obtained from 3 participants (2 due to bleeding and one did not consent for the biopsy). Furthermore, adipose tissue samples from 2 participants were damaged due to early defrosting (adipose specimens were required for collaborators). A total of 52 particpants completed the study.

### RNA yield and characterisation

**Figure 1:**
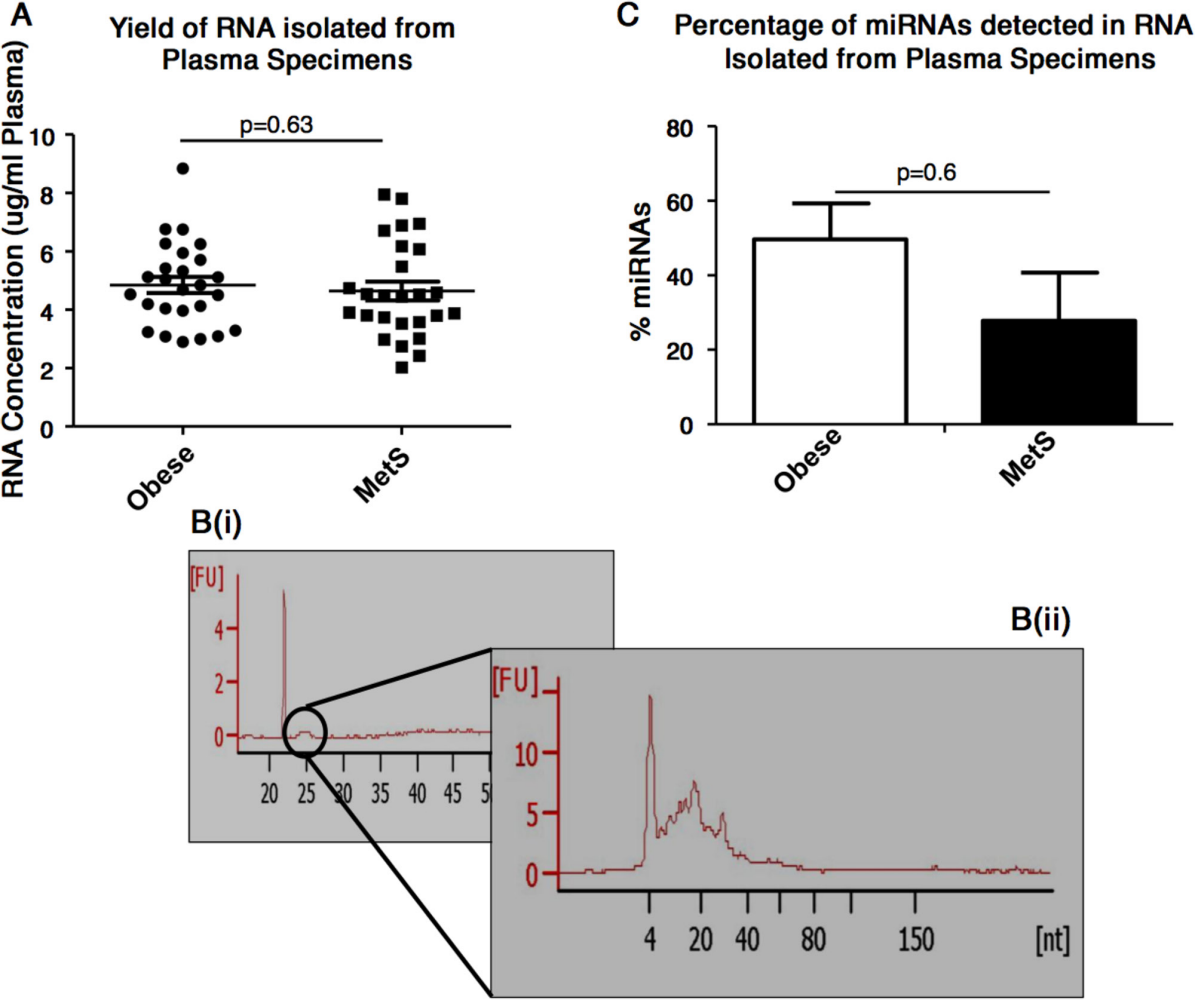
Plasma-based RNA quantification and characterisation **(A)** Concentration of RNA isolated from plasma specimens, as assessed by NanoDrop ND-1000 spectrophotometer. **(B(i))** Electropherograms show the presence of 5S and 5.8S subunits, tRNAs and small RNA fragments (<100bp), but absence of 18S and 28S RNA. **(B(ii))** Electropherograms shows the presence of small RNAs. **(C)** Percentage of miRNAs detected in the total RNA isolated from the plasma of obese and MetS participants.

A similar yield of RNA was obtained from plasma of individuals with obesity and MetS *i.e.* 4.85±0.28 μg RNA/mL plasma for obese individuals and 4.64±0.32 μg RNA/mL plasma for MetS individuals (p=0.63) (Figure 1A). Electropherograms from the RNA 6000 Pico kit analysis of the total RNA showed, as expected, that the plasma specimens do not contains ribosomal RNAs (*i.e.* 18s and 28S were not detected). A small peak was detected at 24-29secs [s]. (Figure 1B(i)). The subsequent small RNA analysis showed this small peak as RNAs of approximately 10-40 nucleotides (Figure 1B(ii)). It also indicated the percentage of miRNAs detected in the total RNA to not differ significantly (p=0.59) between the RNA isolated from plasma of obese and MetS participants (Figure 1C).

### Assessment of miRNAs as potential endogenous control RNAs

For plasma specimens, conventional “endogenous” control RNAs are often not at a constant level as the assessment is not endogenous *per se*; but on extracellular RNA. So here we performed a pre-screen of 172 miRNAs per specimen (for n=20 specimens; including n=10 obese and n=10 MetS) in order to seek RNA(s) that may be suitable as controls for this cohort of specimens. From this, miR-128 (average C_T_=34.2±0.4), miR-150-5p (average C_T_=33.4±0.3), miR-320a (average C_T_=31.6±0.3), miR-486-5p (average C_T_=31±0.5) and U6 snRNA (average C_T_=32.7±0.3) were selected to be included in the custom arrays as potential controls, as all five were detected in all 20 specimens analysed and they were the most uniformly detected miRNAs (average C_T_=32.6±0.3 from all five miRNAs). These five miRNAs were thus included on the custom miRNA array with the 25 miRNAs of interest to test (Table 1). These test miRNAs were selected from a combination of the pre-screen and from literature *e.g.* miR-758-3p was identified as interesting from literature.

**Table 1:** miRNAs selected for inclusion on customised arrays

Selected for Profiling	Reason For Selection from Pre-screen
miR-223-3p	Substantially decreased in MetS vs Obese (−6.6±0.9-fold)
miR-126-3p	>2-fold decreased in MetS vs Obese (−2.29±0.89)
miR-107	>2-fold decreased in MetS vs Obese (−2.51±0.69)
miR-106a-5p	Substantially decreased in MetS vs Obese (−4.56±1.16)
miR-29a-3p	>2-fold decreased in MetS vs Obese (−2.86±0.98-fold)
miR-122-5p	>2-fold increased in MetS vs obese (2.86±0.94-fold)
miR-103a-3p	Substantially decreased in MetS vs Obese (−7.31±1.2)
miR-92a-3p	>2-fold decreased in MetS vs Obese (−2.43±0.80-fold)
miR-106b-5p	Substantially decreased in MetS vs Obese (−7.7±0.95-fold)
let-7d-5p	Substantially decreased in MetS vs Obese (−11.20±0.94-fold)
let-7f-5p	>2-fold decreased in MetS vs Obese (−3.5±0.64-fold)
miR-26b-5p	Substantially decreased in MetS vs Obese (−5.19±0.84)
miR-23a-3p	>2-fold decreased in MetS vs Obese (−3.76±0.79)
miR-23b-3p	>2-fold decreased in MetS vs Obese (−2.76±0.7)
miR-15a-5p	>2-fold decreased in MetS vs Obese (−3.4±1.03)
miR-130a-3p	Substantially decreased in MetS vs Obese (−7.31±1.2)
miR-200c-3p	>2-fold increased in MetS vs Obese (3.72±0.8)
miR-423-5p	Increased in MetS vs Obese (1.6±0.7-fold)
miR-200a-3p	Cited as [31] regulating glucose metabolism
miR-29b-3p	Cited as [32] regulating glucose metabolism
miR-204-5p	Cited as [33] regulating adipocyte differentiation
miR-33a-3p, miR-33b-3p, miR-33b-5p	Cited as [34] regulating lipid metabolism.
miR-758-3p	Cited as [35] regulating cholesterol metabolism
Controls RNAs	miR-128, miR-486-5p, miR-320a, miR-150-5p, U6 snRNA

### miRNAs in plasma specimens from obese and/or MetS individuals

**Figure 2:**
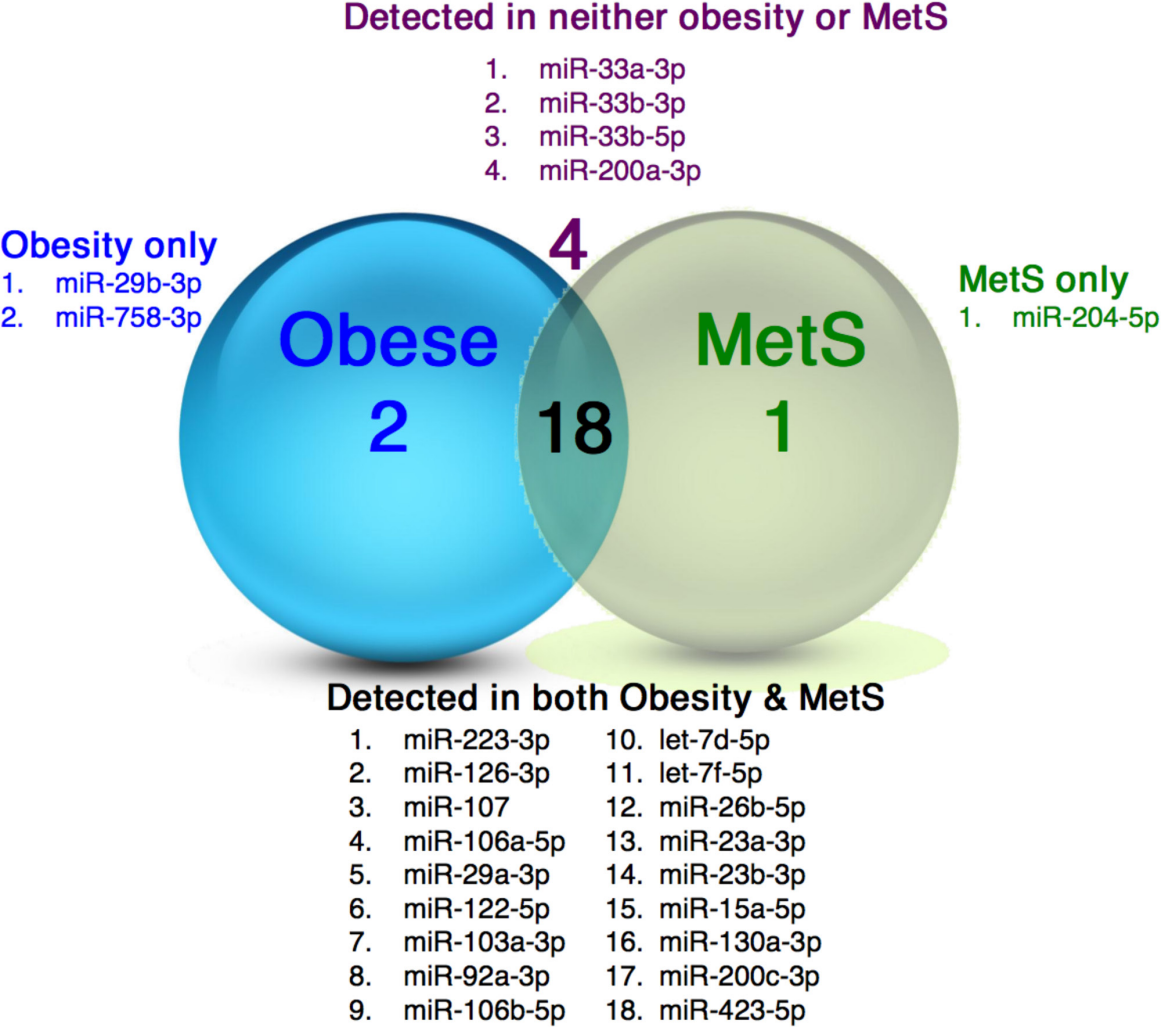
miRNAs in plasma specimens from obese and MetS participants Illustration of the distribution of miRNAs detected in plasma from the obese and MetS individuals.

A cut-off point of 35C_T_ was taken, for all miRNAs, where miRNAs detected ≤35C_T_ were considered as ‘present’ and those with a C_T_ >35C_T_ were considered ‘undetected’. This is frequently done for quantitative polymerase chain reaction (qPCR) data in order to avoid noise from the qPCR analysis. Of the 25 miRNAs analysed, 18 were detected in plasma from both the obese and MetS cohorts, 2 were detected in plasma from the obese cohort only and one was detected in the plasma of the MetS cohort only. Four miRNAs were undetected in both cohorts (Figure 2).

### Identification of potential miRNA biomarkers for MetS - discovery phase

**Figure 3:**
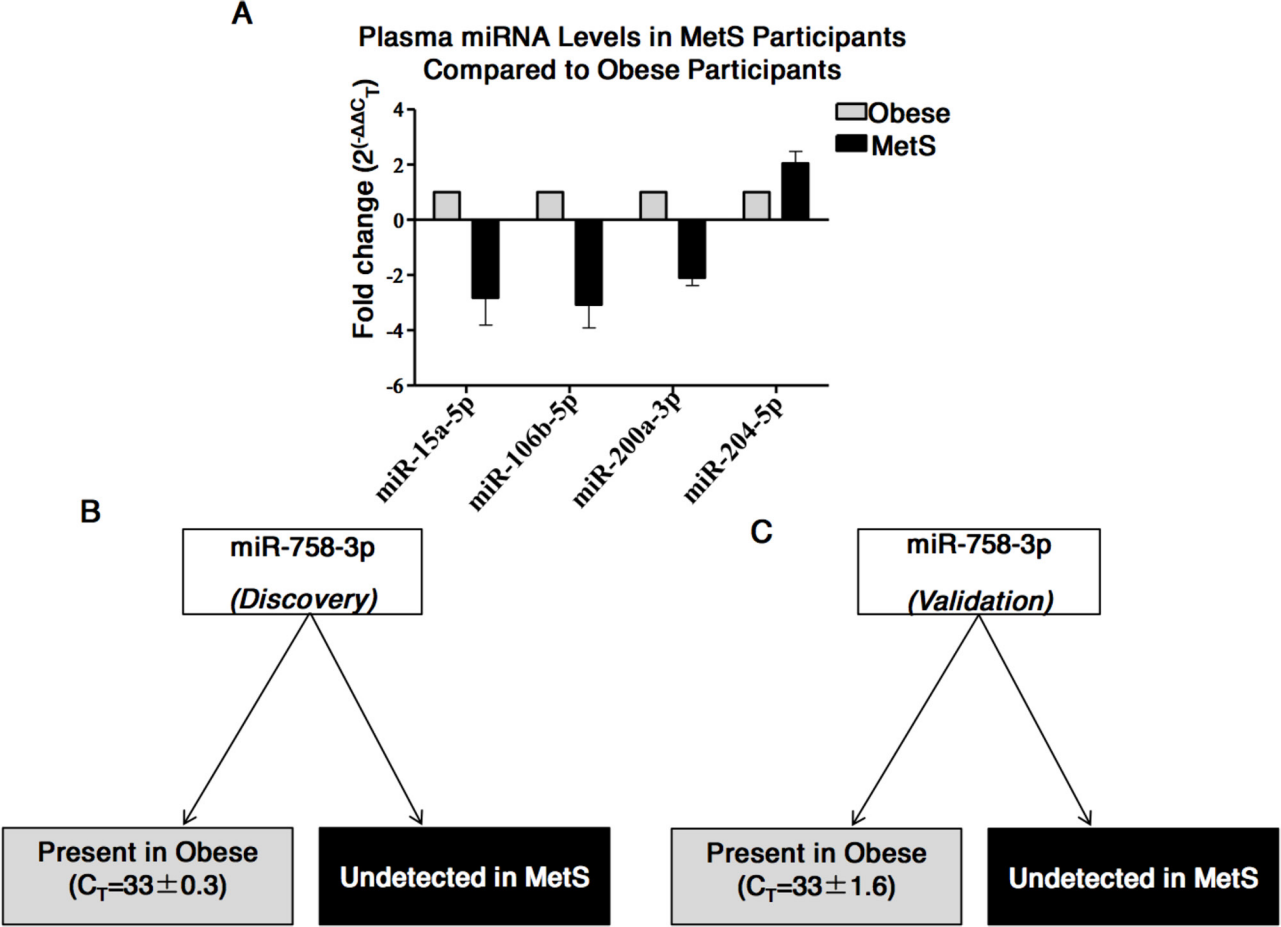
Differences in miRNAs in plasma from MetS compared to Obese participants **(A)** Five miRNAs found to have ≥2-fold difference in quantities in the plasma from MetS compared to obesity. **(B)** miR-758-3p was detected in plasma of obese participants and undetected in the plasma of MetS participants during the discovery stage. **(C)** miR-758-3p detected in plasma of obese participants and undetected in the plasma of MetS participants during the validation stage.

Following normalisation of the customised test miRNA profiling data to the mean C_T_ of miR-150-5p and miR-486-5p (which were by then found to be the most suitable controls), analysis of dysregulated miRNAs between obese (n=19) and MetS (n=19) was performed. Taking a cut-off point of ≥2-fold increased or decreased in specimens from MetS *versus* obese individuals, five miRNAs with potential as biomarkers for MetS were identified. Specifically, the quantities of miR-15a-5p (−2.8±1.4-fold), miR-106b-5p (−3.1±1.2-fold), and miR-200a-3p (−2.1±0.4-fold) were decreased in the MetS cohort and miR-204-5p (2.04±0.6-fold) was increased in the MetS cohort (Figure 3A); although statistical significance was not reached due to person-to-person variability. Interestingly, however, miR-758-3p was detected in all plasma specimens from those with obesity, and was undetected in all plasma specimens from those with MetS (Figure 3B).

### qPCR validation of results arising from discovery phase - validation phase

Individual qPCR assays for miR-15a-5p, miR-106b-5p, miR-200a-3p, miR-204-5p and miR-758-3p in the validation cohort (n=14, 7=obese; 7=MetS) confirmed that four of these five miRNAs were not at significantly different amounts in the MetS cohort compared to the obese cohort. However, miR-758-3p was, again, detected in the obese cohort and undetected in the MetS cohort, validating this result from the discovery phase and suggesting that miR-758-3p may be a biomarker for MetS (Figure 3C).

### Investigation of CERP/ABCA1 as a target of miR-758-3p

**Figure 4:**
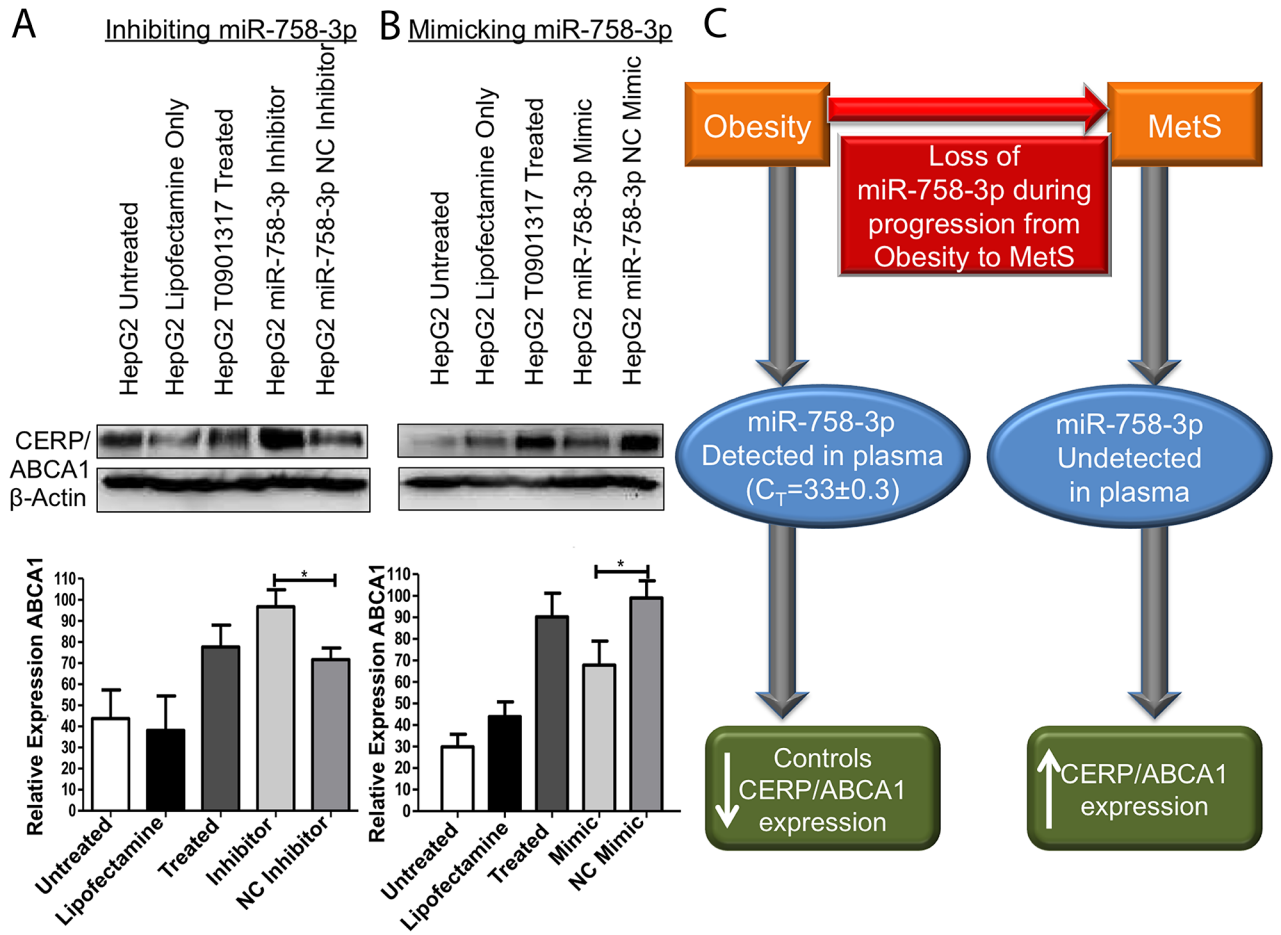
miR-758-3p can control expression of CERP/ABCA1 **(A)** Inhibition of miR-758-3p in HepG2 cells treated with T0901317 (to induce CERP/ABCA1 expression) resulted in a significant (p<0.05) increase in CERP/ABCA1 protein expression compared to ABCA1 levels in negative control (NC) inhibitor transfected cells. **(B)** Conversely, mimicked expression of miR-758-3p in HepG2 cells treated with T0901317 caused a significant reduction in CERP/ABCA1 expression compared to NC mimic transfected cells. Results are shown as representative immunoblots and graphs of densitometry from all immunoblots *i.e.* n=3 ± SEM ^*^p<0.05, ^**^p<0.01, ^**^p<0.001. **(C)** Proposed model of miR-758-3p, which was detected in plasma in obesity and can control CERP/ABCA1 expression, while in MetS the loss of miR-758-3p is associated with increased expression of CERP/ABCA1. Immunoblots were uniformly altered using brightness and contract settings in Powerpoint.

Plasma miR-758-3p demonstrated clinical relevance as a biomarker for progression of obesity to MetS, so our subsequent analysis was to investigate if miR-758-3p can target and control expression of cholesterol efflux regulatory protein/ATP-binding cassette transporter (CERP/ABCA1); its predicted target. As cholesterol efflux occurs in the liver, we selected HepG2 liver cells for further analysis. Here, we used an LXR agonist T0901317 to induce expression ABCA1 in the HepG2 cell line. Inhibition of miR-758-3p resulted in a significant (95% CI: 0.3074-49.70, p=0.0483) increase in the CERP/ABCA1 expression in the HepG2 cell line compared to CERP/ABCA1 expression in negative control inhibitor transfected cells (Figure 4A). Conversely, mimicked expression of miR-758-3p resulted in a significant (95% CI: 3.467-58.98, p=0.0354) decrease in the levels of CERP/ABCA1 compared to negative control mimic transfected cells (Figure 4B). This supports miR-758-3p targeting and controlling CERP/ABCA1 expression in liver cells.

## DISCUSSION

The role of miRNAs and their potential as biomarkers for diseases such as cancer has long been investigated by ourselves [16–18] and others [19, 20]. Here, we progressed to investigate the potential use of blood-based miRNAs as biomarkers for metabolic syndrome (MetS) and the putative ability of miRNAs in the peripheral circulation to discriminate progression from obesity to MetS [3]. To the best of our knowledge, this is the first examination of the differences in blood-based miRNAs in MetS individuals compared to obese individuals.

Our study was designed in such a way as to only include blood plasma specimens from individuals who were being selected to subsequently take part in a randomised, parallel controlled, double-blinded, dietary intervention study (DairyHealth [21]). This study design was to ensure comprehensive prior clinical assessment of the individuals, so as to accurately characterise them as clinically obese or MetS; to fully adhere to the inclusion and exclusions criteria described here; to only procure blood specimens from individuals in a fasting state and in the morning; and to process all specimens using standard operation procedures performed by appropriately trained personnel. The reason for applying this level of stringency was to avoid, in as far as at all possible, noise in data and differences in results that can occur in biomarker studies that are not designed prospectively. As a simple example, if participants donated blood specimens at various times throughout the day, some before and some after meals, there are increased chances of differences in circulating miRNAs being at least partially due to effects of these variables and not more specifically due to biological differences between obesity and MetS. Additionally, these plasma specimens are limited in volume (as they are part of an intervention study), as many other baseline parameters had to be assessed [21, 22]. Thus, we elected to perform a pre-screen on a limited number (n=20) of specimens. We also considered key miRNAs from literature mining. This was to inform a rationally-designed focussed analysis of 25 miRNAs, rather than what is sometime described as a “fishing trip” of many hundreds of miRNAs but on fewer specimens. For example miR-204-5p was found to be significantly increased in the MetS cohort (10 specimens) compared to the obese cohort (10 specimens) following the pre-screen. This was interesting as miR-204-5p has been shown to be increase during adipogenesis [4].

Of note, efforts were also made to identify control miRNAs against which to normalise our data, as we [17] and others working in the field of blood-based miRNAs have previously reported. Serum/plasma studies do not involve miRNAs that are actually intracellular/endogenous and to date no single blood-based miRNA that suits all study comparisons has been identified. However, in these particular conditions *i.e.* obesity and MetS, we found the mean levels of a number of miRNAs most suitable (rather than a single miRNA) as control miRNAs for normalising our data. Furthermore, with plasma specimens procured from 52 individuals (26 with MetS, 26 with obesity) one option we had was to perform only a “discovery” study *i.e.* only investigate differential miRNA detection, with the hope of attempting an initial validation in the future. However, when designing the study –with all clinicians, statisticians and scientists in agreement– a more meaningful and useful study, for ourselves and others to build upon, would include a discovery and a validation stage.

Using this approach of a random discovery cohort and validation cohort, we identified miR-758-3p as a potential biomarker for discriminating MetS from obesity *i.e.* miR-758-3p was undetected in plasma from individuals in the MetS cohort but was detected in plasma from all individuals in the obese cohort. We believe that further studies to independently validate these finding are now warranted.

To understand if the loss of miR-758-3p in progression from obesity to MetS may reflect pathophysiological relevance, we then investigated the potential targets of miR-758-3p using miRNA-target predictor miR-walk, miR-Walk collates predictions from DIANAmT, miRanda, miRDB, miR-Walk, RNAhybrid, PICTAR4, PICTAR5, PITA, RNA22 and Targetscan. Here, cholesterol efflux regulatory protein/ATP-binding cassette transporter A1 (*i.e.* CERP/ABCA1) was predicted as the target of miR-758-3p. CERP/ABCA1 is a transmembrane transporter [23] that, for many years, has been well established as a key mediator of cholesterol homeostasis, facilitating cellular free cholesterol efflux to an extracellular acceptor (apolipoprotein AI, apoA-I) to form nascent high density lipoprotein HDL [24, 25]. Approximately 70% of nascent HDL is generated in the liver when Apo-AI, which is generated by hepatocytes, interacts with hepatic CERP/ABCA1 [26]. Interestingly, in a recent study of CERP/ABCA1 cholesterol efflux in serum from people with MetS and healthy controls, MetS patients were found to present significantly higher CERP/ABCA1 mediated cholesterol efflux than their healthy counterparts. To accept cholesterol from cholesterol-loaded BHK cells expressing either ABCA1 or ABCG1 [27].

As CERP/ABCA1 has been shown to be responsible for inducing formation of HDL in the liver, to confirm our hypothesis that loss of miR-758-3p may reflect pathophysiological relevance, we selected the liver cell line HepG2 for subsequent functional studies. Using both mimics and inhibitors, in keeping with the work of Ramirez *et al.* [28] and Lucero *et al.* [27], we confirmed that loss of miR-758-3p (as we observed in MetS) resulted in increased expression of CERP/ABCA1 on the liver cells. This may be the mechanism responsible for the significantly higher CERP/ABCA1 mediated cholesterol efflux reported in MetS [27]. Conversely, we found miR-758-3p mimics to have the opposite effect on CERP/ABCA1 *i.e.* to reduce CERP/ABCA1 expression, which was made more difficult to achieve given that the use of an LXR agonist to induce ABCA1 would reduce the efficacy of the miR-758-3p mimic. Taken together, this suggests that modulating miR-758-3p may have therapeutic relevance in conditions contributed to by too little or too much miR-758-3p expression; such as MetS.

In conclusion, our study shows for the first time that miR-758-3p holds potential as a minimally-invasive blood-based biomarker for aiding the diagnosis of MetS, by distinguishing MetS individuals from those with clinical obesity. Efforts towards progressing miR-758-3p to the clinic for use as a diagnostic biomarker would, of course, involve the validation of the results shown here in independent cohorts of participants. Considering the potential pathophysiological relevance of this loss of miR-758-3p in the progression of obesity to MetS, we also provide functional evidence that miR-758-3p targets CERP/ABCA1 in liver cells, in keeping with the reported higher CERP/ABCA1 mediated cholesterol efflux in MetS (in that case, compared to healthy controls). Identification of this miRNA as a potential blood-based biomarker for MetS –and possibly also causally involved in these events– may help address an unmet need, as the societal and economic burden of obesity progressing to MetS and the subsequent increased risk of CVD, T2DM and cancer is substantial. Further independent assessment of miR-758-3p and its control of CERP/ABCA1 –both as a diagnostic biomarker and a therapeutic intervention– is now warranted. It would also be very interesting to establish if the miRNAs detected are associated with RNA-binding protein in the sera or if they are associated with extracellular vesicles.

## MATERIALS AND METHODS

### Study design and participant plasma specimens

63 individuals were recruited to DairyHealth (NCT01472666) in the Department of Endocrinology and Internal Medicine Aarhus University, Denmark between October 2011 and December 2012 through local newspapers and electronic advertisement [21]. The number of participants needed to complete the study and achieve a statistical power of 80% was calculated to be 52 (α < 0.05, β = 0.80). Fifty-two individuals including 26 individuals who were diagnosed with MetS according to the IDF definition (female: n=14, 59.5±3.2 yrs; male: n=12, 58.6±3.8 yrs) and 26 individuals who were clinically obese (female: n=13, 47.1±5.2 yrs; male: n=13, 61.5±2.9 yrs) completed the study (see Table 2 for participant characteristics). Participants were screened on the basis of their medical history and a physical examination. Inclusion criteria included ≥18 years; abdominal circumference of ≥90cm (males) and ≥80cm (females); weight stable (±5kg) for at least 3 months prior to inclusion. Exclusion criteria included T2DM; severe cardiovascular, renal, endocrine or psychiatric disease; drug abuse; pregnancy or lactation; and blood donation was prohibited for 3 months prior to inclusion.

**Table 2:** Participant characteristics

Participant Characteristics	Male	Female
Age (yrs±SEM)	56.7±2.03	56.3±2.14
MetS (n)	12	14
Obese (n)	13	13
BMI (kg/m^2^)±SEM	28.8±0.51	30.2±4.4
Anti-hypertensive treatment	6 (24%)	3 (11.1%)
Cholesterol lowering treatment	5 (20%)	6 (22.2%)

Upon arrival at the clinic in the morning, individuals were fasting and a blood specimen was procured, plasma was isolated and stored at −80^°^C for subsequent analysis. The study was designed to include a two-stage analysis of miRNAs present in the plasma *i.e.* a discovery stage, using a customised array for a total of 38 specimens, including specimens from 19 individuals with obesity and 19 individuals with MetS and being mindful to balance male and female participants within these sub-cohorts, but otherwise selected randomly. This was followed by a validation stage, by qPCR, for the remaining 14 specimens (n=7 obesity; n=7 MetS). The study protocol was performed in accordance with the Helsinki Declaration of 1975 as revised in 1983 and was approved by the Central Denmark Region Committees on Health Research Ethics. The study was registered with clinicaltrials.gov as NCT01472666. Additionally, after receiving written and oral information, all participants gave their written informed consent before participating in the study.

### RNA extraction and characterisation

RNA was isolated from 250 μL of each of the plasma specimens. Specifically, RNA was extracted using TriReagent (Sigma-Aldrich, T9424) using a modified procedure that we had previously reported [29]. In brief, 200 μL chloroform (Sigma-Aldrich, C424) was added to each specimen, followed by a 15 min incubation, centrifugation and retention of the aqueous phase. Glycogen (Sigma, G8751) (final concentration 120 μg/mL) was used as a carrier and 500 μL isopropanol (Sigma, I9516) was added to precipitate out the RNA. RNA quantity was subsequently assessed using a NanoDrop ND-1000 (Labtech International, Uckfield, England) and Agilent Pico 6000 and Small RNA kits (Agilent Technologies, CA, USA) as per manufacturers’ instructions.

### miRNA profiling

Prior to initiating the discovery phase, efforts were made to identify one or more controls that may be unchanged in all specimens. This involved using Exiqon’s Focus Panel (Exiqon, 203843) consisting of 172 miRNAs and 7 plate control RNAs for 20 specimens. A customer-designed Exiqon pick-and-mix panel was subsequently generated as the array platform for profiling (Exiqon, 203815) for use with the ABI-7900HT Sequence Detection System (Applied Biosystems, Foster City, CA, USA). Twenty-five miRNAs and a further five sequences identified as potential controls, selected from a combination of the Focus Panel and informed by published literature (Table 1), were placed on the customised arrays along with UniSp3IPC and UniSp6 technical control

### Reverse transcription (cDNA synthesis) discovery miRNA profiling

Reverse transcription (RT) of miRNAs to cDNA was performed according to the Exiqon miCURY LNA^TM^ Universal RT miRNA PCR instruction manual for serum/plasma and other biofluids samples. Briefly, RNA (suspended in 1.6 μL of nuclease-free water) for each specimen was added to 2 μL of 5X reaction buffer, 1 μL of enzyme mix, 4.9 μL of nuclease-free water and 0.5 μL of UniSp6 spike-in. cDNA was synthesised using the Applied Biosystems Veriti 96 well thermal cycler (Applied Biosystems, Foster City, CA, USA) as follows: incubate at 42°C for 60 mins, heat-inactivate the reverse transcriptase at 95°C for 5 mins and cool to 4°C.

### qPCR

qPCR was performed also according to the Exiqon miCURY LNA^TM^ Universal RT miRNA PCR instruction manual for serum/plasma and other biofluid samples. As the pre-screen showed miR-486-5p and miR-150-5p quantities do not differ significantly between specimens, these were considered as controls. C_T_ values for other miRNAs were normalised to the mean quantity of miR-486-5p plus miR-150-5p. qPCR was performed using the ABI-7900HT Sequence Detection System with the conditions as recommended by Exiqon *i.e.* hold at 95°C for 10 mins; qPCR stage at 95°C for 10 secs and 60°C for 1 min, by 40 cycles and melt stage at 95°C for 15 secs, 60°C for 1 min, 95°C for 15 secs.

### Validation of potential biomarkers for MetS

Subsequent efforts to validate 5 miRNAs selected from the discovery stage were performed using sequence-specific primers *i.e*. miR-15a-5p (Exiqon, 204066), miR-106b-5p (Exiqon, 205884), miR-200a-3p (Exiqon, 204707), miR-204-5p (Exiqon, 205708) and miR-758-3p (Exiqon, 204353). Again, miR-486-5p (Exiqon, 204001) and miR-150-5p (Exiqon, 204660) were included as controls, with UniSp6 as technical control.

### Data analysis

ABI SDS v2.4 software was used to obtain C_T_ values. The raw data was exported using SDS software and UnSP3IPC was used to calibrate for differences in plate runs using the equation: Specimen C_T_- ((average of UniSP3IPC for the plate)-(average of UniSp3IPC for all plates)). UniSp6 confirmed the RT and PCR reactions were successful; therefore any miRNAs not detected in the study was not due to technical error. Fold changes of miRNAs in MetS specimens versus obese specimens were subsequently determined by the ΔΔC_T_method.

### Bioinformatics analysis

miR-Walk, collating data from a number of prediction softwares, including DIANAmT, miRanda, miRDB, miR-Walk, RNAhybrid, PICTAR4, PICTAR5, PITA, RNA22 and Targetscan, was used to identify potential targets of miRNAs. From this analysis, the cholesterol efflux regulatory protein/ATP-binding cassette transporter (CERP/ABCA1) was predicted as a miR-758-3p target.

### Cell culture

HepG2 (human liver carcinoma) [30] cells were obtained from ATCC. Cells were authenticated using short tandem repeat profiling (May, 2016) and cultured in Eagle’s MEM (Sigma-Aldrich, M2279) containing 10% fetal bovine serum (Gibco, 10270106), 2mM L-Glutamine (Sigma-Aldrich, I0516) and 1X non-essential amino acids (Sigma-Aldrich, M7145) at 37°C with 5% CO_2_.

### miRNA inhibition/mimic manipulation in HepG2 cells

HepG2 cells seeded at 4×10^5^ cells/well in a 6-well plate were transfected, using lipofectamine 2000 (Invitrogen, 11668-027), with miR-758-3p inhibitor (Dharmacon, 4100323-021), miRNA inhibitor negative control (Dharmacon, 12610636), miR-758-3p mimic (Dharmacon, 15475264) or miRNA mimic negative control (Dharmacon, 11587170); each at a final concentration of 40 nM. 24 hrs post-transfection, cells were treated with LXR agonist T0901317 (Sigma-Aldrich, T2320) to induce CERP/ABCA1 expression. 24 hours later cells were harvested for protein isolation and immunoblotting.

### Immunoblotting

Total protein (50 μg) was resolved on 7% SDS-PAGE and transferred to PVDF membranes (Bio-Rad Laboratories, 162-0177). Primary antibodies to CERP/ABCA1 (Abcam, 18180, 1:200, mouse monoclonal antibody) and β-actin (Sigma-Aldrich, A5316, 1:1000, mouse monoclonal antibody) were used. Following incubation with the appropriate anti-mouse horseradish peroxidase-conjugated secondary antibodies (Cell Signalling, 7076s, 1:1000, horse anti-mouse IgG antibody), immunoblots were developed using chemiluminescence (Thermo Fisher, 34080) and detected on a Chemidoc exposure system (Bio-Rad Laboratories, Hercules, CA, USA). Densitometry was performed using NIH ImageJ software and CERP/ABCA1 was normalised to the loading control β-actin.

### Statistical analysis

Statistical analysis was performed in Excel. Fold changes between miRNAs detected in the MetS cohort compared to miRNAs detected in the obese cohort were calculated using the ΔΔC_T_ method. Specifically, ΔC_T_=Each miRNA C_T_ value-average C_T_ value of control miRNAs; ΔΔC_T_=miRNA C_T_ value in MetS cohort-miRNA in obese cohort C_T_ value; fold change=ΔΔC_T_ plugged into formula 2^(−ΔΔC^_T_). P-values were generated using Student’s *T*-tests, with p<0.05 considered as statistically significant. GraphPad Prism 5.0 was used for graph generation (Graph Pad Software Inc, La Jolla, CA, USA). Analysis of the raw data was performed both prior to and following controlling for clinical data available for all participants (Table 2). However, no difference prior to and after controlling for these parameters was observed.

## Abbreviations

CERP/ABCA1: cholesterol efflux regulatory protein/ATP-binding cassette transporter
CT: cycle threshold
MetS: metabolic syndrome
IDF: International Diabetes Federation
WHO: World Health Organisation
NCEP:ATPIII: National Cholesterol Education Program: Third Adult Treatment Panel
T2DM: Type 2 Diabetes Mellitus
CVD: cardiovascular disease
Apo-A1: Apolipoprotein A1.
